# Supplementary material for: Human RTEL1 Interacts with KPNB1 (Importin β) and NUP153 and Connects Nuclear Import to Nuclear Envelope Stability in S-Phase
Source: Cells. 2023 Dec 8;12(24):2798. doi: 10.3390/cells12242798 (PMC10741959; doi:10.3390/cells12242798)

Article

Human RTEL1 interacts with (KPNB1) (importin β) and NUP153 and connects nuclear import to nuclear envelope stability in S-phase

Michael Schertzer[1,2], Laurent Jullien[3,4], André L. Pinto[5], Rodrigo T. Calado[5], Patrick Revy[3,4] and Arturo Londoño-Vallejo[1,2*]

LEGENDS TO SUPPLEMENTARY FIGURES

Supplementary Figure S1

A-B. Immortalized HHS patient and normal dermal fibroblasts (+SV40-LT, +hTERT) were treated for IF using anti-LMNB1 anti-LMNA/C antibodies and counter-stained for DAPI before circularity of nuclei was measured. **C**. Whole cell extracts from immortalized HHS fibroblasts were analyzed by western blot for LMNA/C, LMNB1, LMNB2 and histone H4. Ponceau staining of the membrane is also shown for protein loading comparisons. **D**. Primary dermal fibroblasts from a Dyskeratosis congenita patient with a heterozygous RTEL1 mutation in the proximal harmonin repeat (Y1086C), along with immortalized normal and HHS patient dermal fibroblasts (+SV40-LT, +hTERT) were treated for IF using anti-LMNB1 and anti-LMNA/C antibodies and counter-stained for DAPI. **E**. Western blot protein analysis (whole cell extracts) for RTEL1 in HeLa cells treated with either CRISPR Cas9 IRES GFP targeting RTEL1 gene or a scrambled sgRNA and corresponding to the experiment described in Figure 2A. PCNA and Ponceau staining are also shown for protein loading. Molecular weights in kDa are indicated. **F**. Asynchronously growing immortalized HHS fibroblasts in culture or HeLa cells expressing RTEL1-MR were treated with EdU for 15 minutes. After pre-extraction and PFA fixation, EdU was detected with Click-it 488 chemistry and nuclei were counterstained with DAPI. **G,H**. Experimental schematics for induction of RTEL1 during synchronization using a double thymidine block. Tetracycline is added during the second round of thymidine treatment. Cells are released into S-phase 18h later and cells were fixed at 0h, 3h and 6h after release (in all subsequent experiments cells were released for 1h), plated on a slide and counter-stained with DAPI. Nuclei were visualized under the microscope using a 40x objective and counted. The percentage of aberrantly shaped nuclei was scored. FACS analysis of BrdU incorporation measured against DAPI content in HeLa cells is shown as validation for the cell cycle block. **I,J**. Synchronized HeLa cells either uninduced or induced for the expression of either wild type RTEL1, or RTEL1-MR were blocked at G1/S or released 1h after the block. Cells were fixed and then IF was done with anti-LMNA/C and counter-stained with DAPI before circularity of nuclei was measured. **B,D,F,I**. Scale bars are 5μm. **A,J**. Statistical comparisons were done using a Mann-Whitney test (****=p<0.0001) and the number of cells analyzed is indicated.

Supplementary figure S2

A. Synchronized HeLa cells, blocked at G1/S and either uninduced or induced for the expression of an RTEL1 version mutated in the NLS or the RING were released into S-phase. Cells were fixed and then IF was carried out with anti-LMNA/C and anti-RTEL1 antibodies and counter-stained with DAPI before circularity of nuclei was measured (see Figure 3E). **B**. Synchronized HeLa cells, uninduced or induced with Tetracycline for the expression of the indicated RTEL1 proteins were pre-extracted (0.5% Triton and 50mM NaCl) before PFA fixation and then IF was done with anti-RTEL1 and counter-stained with

DAPI. **C**. Western blot protein analysis (whole cell extracts) for RTEL1 in uniduced HeLa cells or cells induced with Tetracycline for the expression of NLSmut. Ponceau staining are also shown for protein loading and molecular weights in kDa are indicated. **A,B**. Scale bars are 5μm.

Supplementary Figure S3

A. Synchronized HeLa cells either uninduced or induced for the expression of either wild type RTEL1, RTEL1-MR or NLSmut were blocked at G1/S and treated with importazole (40μM) or ivermectin (50μM) for 3h. Cells were fixed before release or released for 1 hour in S-phase and then treated for IF with anti-RTEL1 and counter-stained with DAPI before nuclear fluorescence for RTEL1 was measured. **B**. Quantifications of relative amounts of RTEL1 in the nucleus/total in cells treated or not with importazole (+imp) in A. **C**. Quantifications of relative amounts of RTEL1 in the nucleus/total in cells treated or not with ivermectin (+ IVM, 50μM for 3h) in A. **B,C**. Statistical comparisons were done using a Mann-Whitney test (****=p<0.0001) and the number of cells analyzed is indicated. **D**. FLAG-RTEL1 or empty vector were transfected into 293T cells for 24h. Lysates were treated for immunoprecipitation with an anti-FLAG antibody and analyzed by Western Blot using anti-RTEL1 or anti-KPNB1. Non-specific immunoglobulin band indicated by (*). **E**. A vector expressing wild type RTEL1 was co-transfected with FLAG-tagged N-terminal fragments of KPNB1 (positions 1-462 or 1-296) or with the FLAG vector into 293T cells for 24h. Lysates were treated for immunoprecipitation with an anti-FLAG antibody and analyzed by Western Blot using anti-RTEL1, anti-RAN or anti-FLAG antibodies. Positions of known interacting regions for RAN and importin α (KPNA2) are indicated. Non-specific immunoglobulin band indicated by (*). **F**. Vectors expressing different RTEL1 fragments as illustrated were co-transfected with a FLAG-tagged N-terminal fragment of KPNB1 (1-462) or with the FLAG vector into 293T cells for 24h. Lysates were treated for immunoprecipitation with an anti-FLAG antibody and analyzed by Western Blot using anti-RTEL1 and anti-FLAG antibodies. A representation of RTEL1 indicating known functional domains and those identified in this study (in green) and the RTEL1 proteins used for the co-transfections and their terminal amino acid positions are indicated. **D-F**. Molecular weights in kDa are indicated.

Supplementary Figure S4

A. Synchronized HeLa cells either uninduced or induced for the expression of RTEL1 C-terminal fragments (the most N-terminal position for each construction is indicated) lacking the helicase domain and fused to GFP were blocked at G1/S and treated (or not - UT) with importazole for 3h (40μM). The 683-NLSmut-1300 carries mutations abolishing the classic import pathway. Cells were fixed before IF and stained with anti-LMNA/C antibodies and counter-stained with DAPI and nuclear circularity was measured (see Figure 5B for quantifications). The GFP signal corresponding to the fused versions of RTEL1 was directly visualized on the green channel (with exception of HeLa where an anti-RTEL1 antibody was used). **B**. Synchronized HeLa cells induced for the expression of RTEL1 C-terminal fragments (the most N-terminal positions are indicated) lacking the helicase domain, fused to GFP and carrying (or not) mutations in the RING domain were blocked at G1/S, and then released into S-phase for 1h. Cells were fixed, treated for IF with anti-LMNA/C antibodies and counter-stained with DAPI and nuclear circularity was measured (see Figure 5C for quantifications). The GFP signal corresponding to the fused versions of RTEL1 was directly visualized on the green channel. **C**. Whole cell extracts from stably transfected Hela cell lines, induced (+Tet) or not for the expression RTEL1 C-terminal fragments (the most N-terminal positions are indicated) lacking the helicase domain and fused to GFP (or not), were analyzed by Western blot using an anti-RTEL1 antibody. Molecular weights in kDa are indicated.

D,E. Synchronized HeLa cells induced (or not) for the expression of GFP fusions of RTEL1 C-terminal fragments (positions are indicated) lacking the helicase domain and carrying mutations in the NLS (when indicated NLSmut, GFP fused to the ITD (683-808) or GFP alone, were blocked at G1/S, and then released into S-phase for 1h. Cells were fixed, treated for IF with anti-LMNA/C antibodies and counter-stained with DAPI before examination under the microscope. GFP signal was directly visualized on the green channel and its intensity measured to calculate nuclear enrichment as above (see figure 5D). Cells were fixed and counter-stained with DAPI. The GFP signal corresponding to the fused versions of RTEL1 was directly visualized on the green channel and its relative Nuclear/Total intensity calculated. **F**. Synchronized HeLa cells induced for the expression of the 683-808 domain of RTEL1 (ITD) fused to GFP were blocked at G1/S and treated (or not - UT) with importazole for 3h (40μM). Cells were fixed, treated for IF with anti-LMNA/C antibodies and counter-stained with DAPI. The GFP signal corresponding to the fused ITD was directly visualized on the green channel and its relative Nuclear/Total intensity calculated. (see figure 5E for quantifications). **A,B,F**. Scale bars are 5μm. **D,E**. Statistical comparisons were done using a Mann-Whitney test (**=p<0.01; ****=p<0.0001) and the number of cells analyzed is indicated.

Supplementary Figure S5

A. 293T cells were transiently transfected with GFP-tagged N-terminal fragments of NUP153 (positions are indicated) and lysates were treated for immunoprecipitation with an anti-GFP antibody and analyzed by Western blot with both anti-GFP and anti-NUP50 antibodies. **B**. 293T cells were transiently co-transfected with GFP-tagged N-terminal fragment of NUP153 (1-434) and either empty FLAG vector, FLAG-tagged full length RTEL1, or C-terminal RTEL1 (683-1300). Lysates were treated for immunoprecipitation with an anti-FLAG antibody and analyzed by Western blot with both anti-GFP and anti-FLAG antibodies. **C**. 293T cells were transiently transfected with GFP or GFP-RTEL1-683-808 and lysates were treated for immunoprecipitation with an anti-GFP antibody. Western blot analysis of immunoprecipitates was done with anti-GFP and nuclear pore components anti-NUP153, anti-NUP62, anti-RANBP2, and anti-NUP98. **D**. Fluorescent image using a ZOE flourescent cell imager (BioRad) of transfected 293T cells used for the immunoprecipitation in supplemental 5C; scale bar 100μm. **A-C**. Molecular weights in kDa are indicated.

Supplementary Figure S6

A. Multi-species amino acid sequence alignment for RTEL1 ITD and the domain that interacts with importin β. Positions in the human sequence are indicated. The beginning of the 1178-1210 includes the highly conserved PIP box. The red box highlights highly conserved residues amongst hominins. * identical residue, : conserved residue, . semi-conserved residue.

Supplementary Figure S1

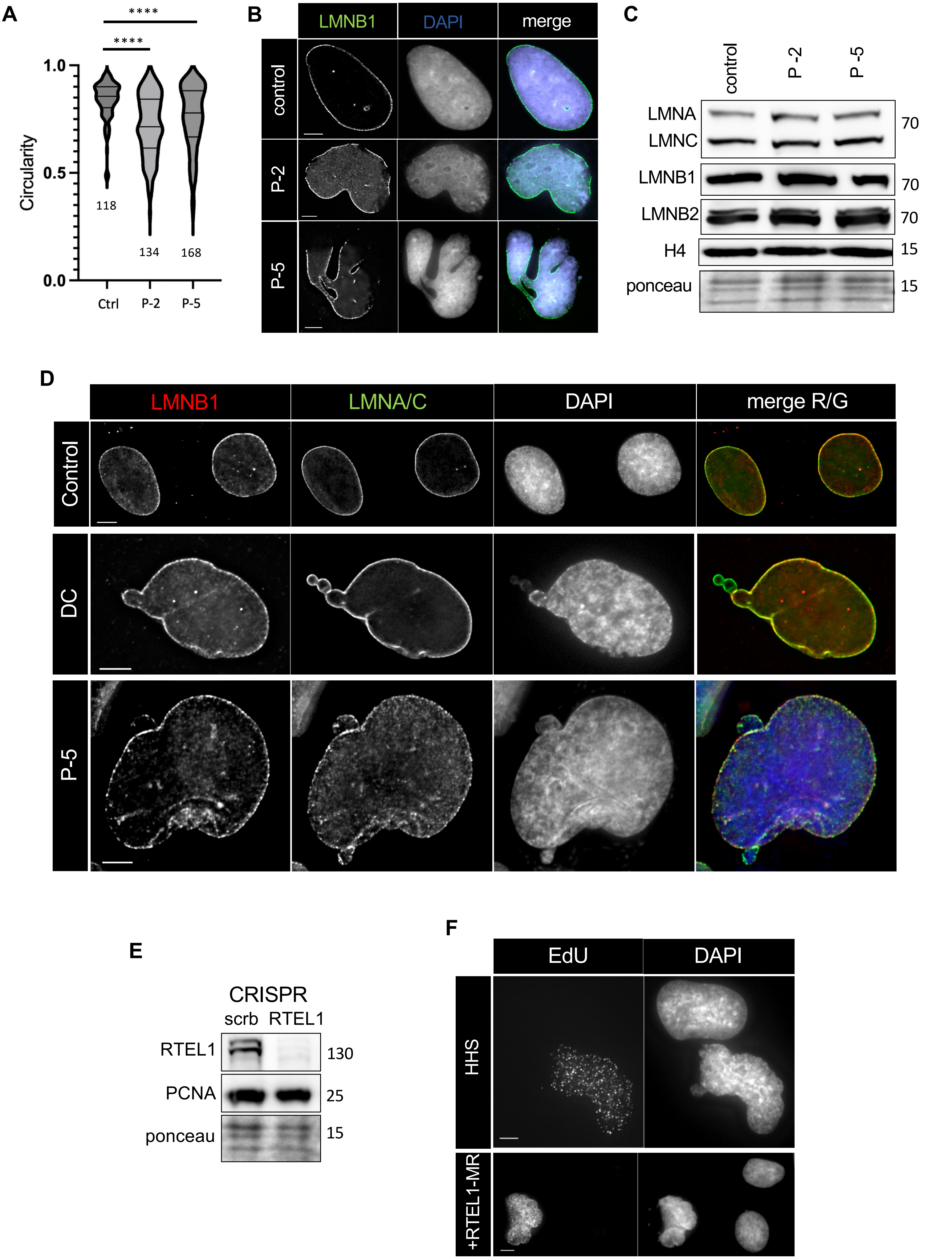

Supplementary Figure S1 (cont…)

G

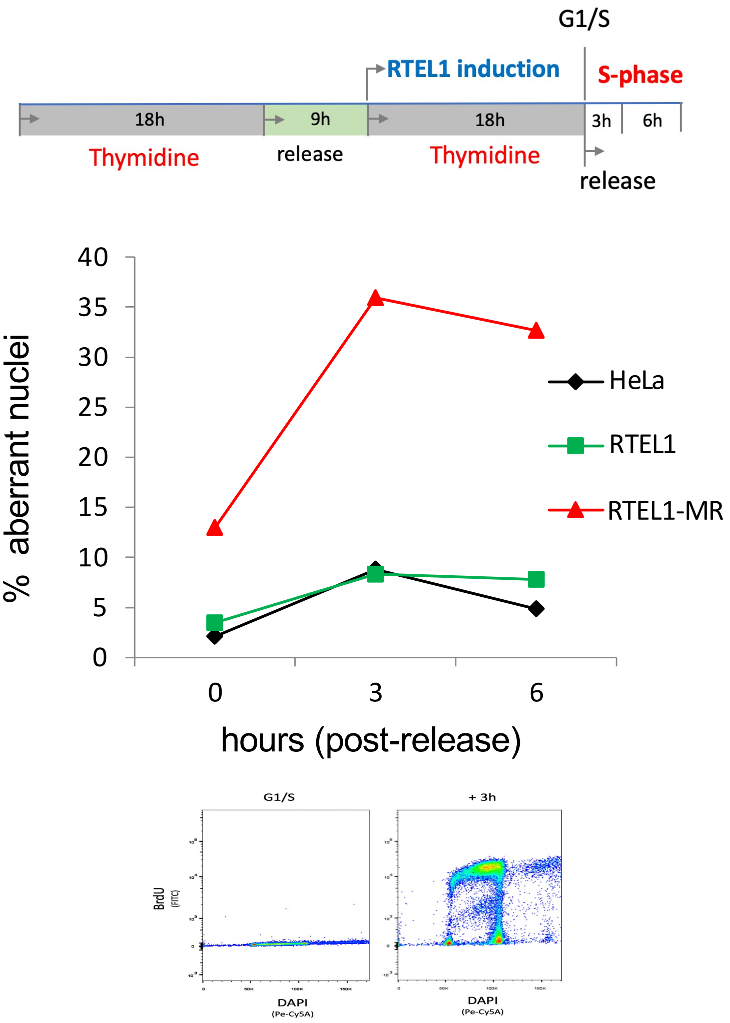

H

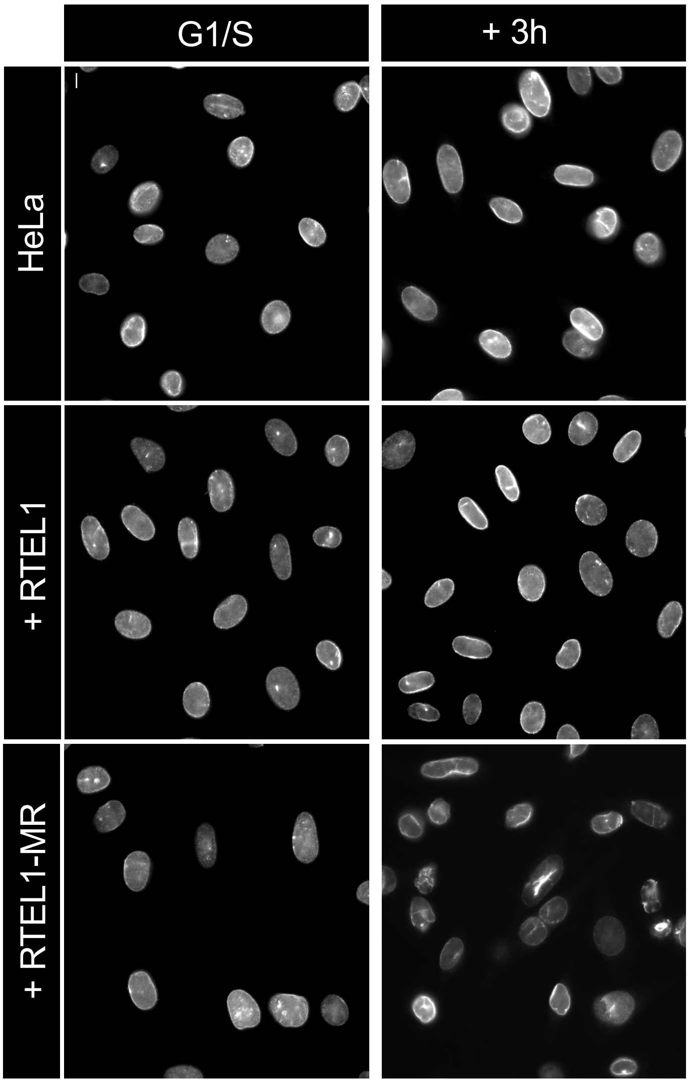

I

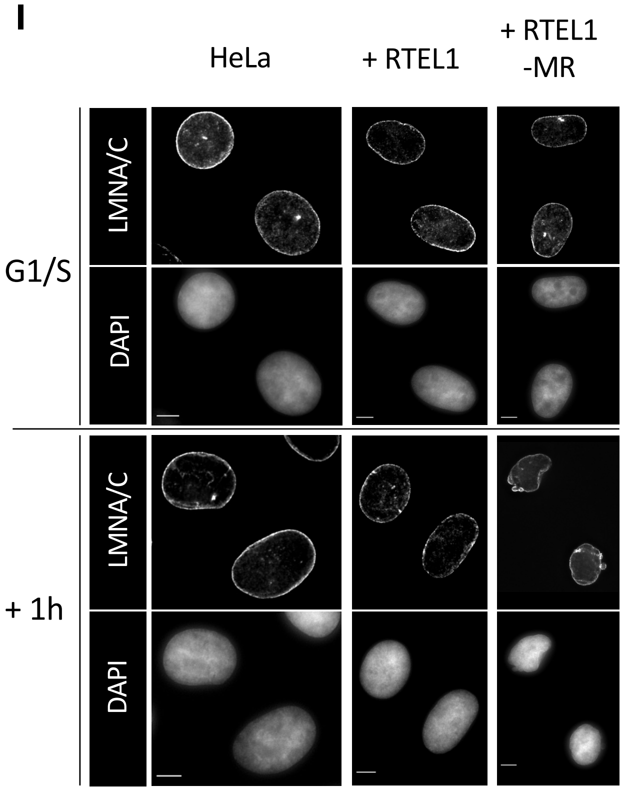

J

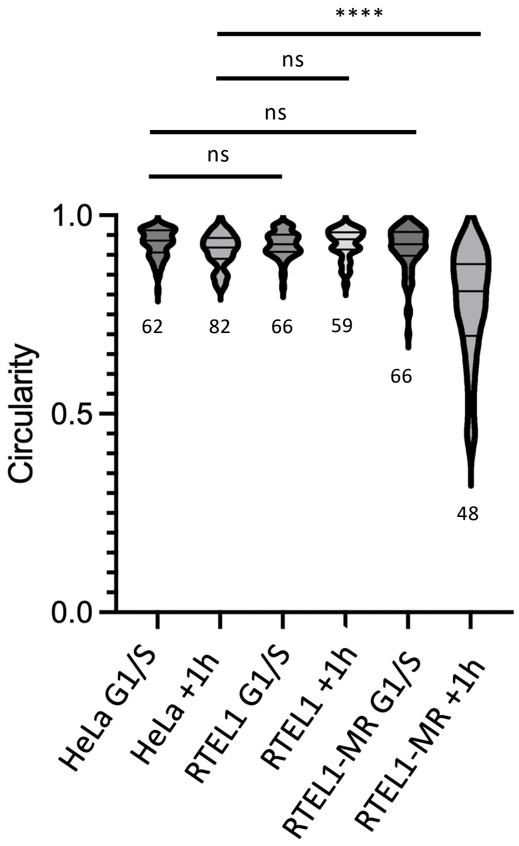

Supplementary Figure S2

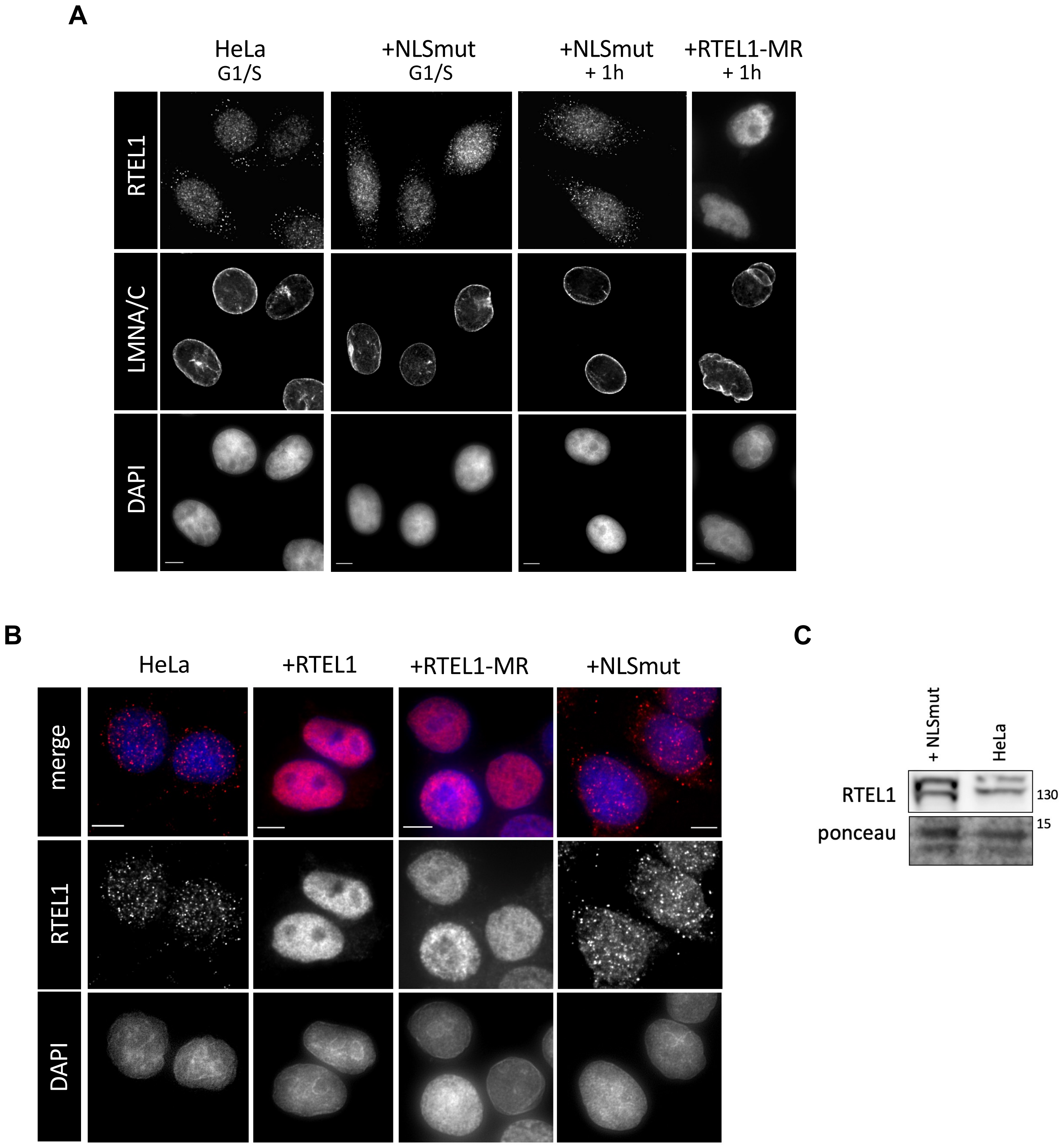

Supplementary Figure S3

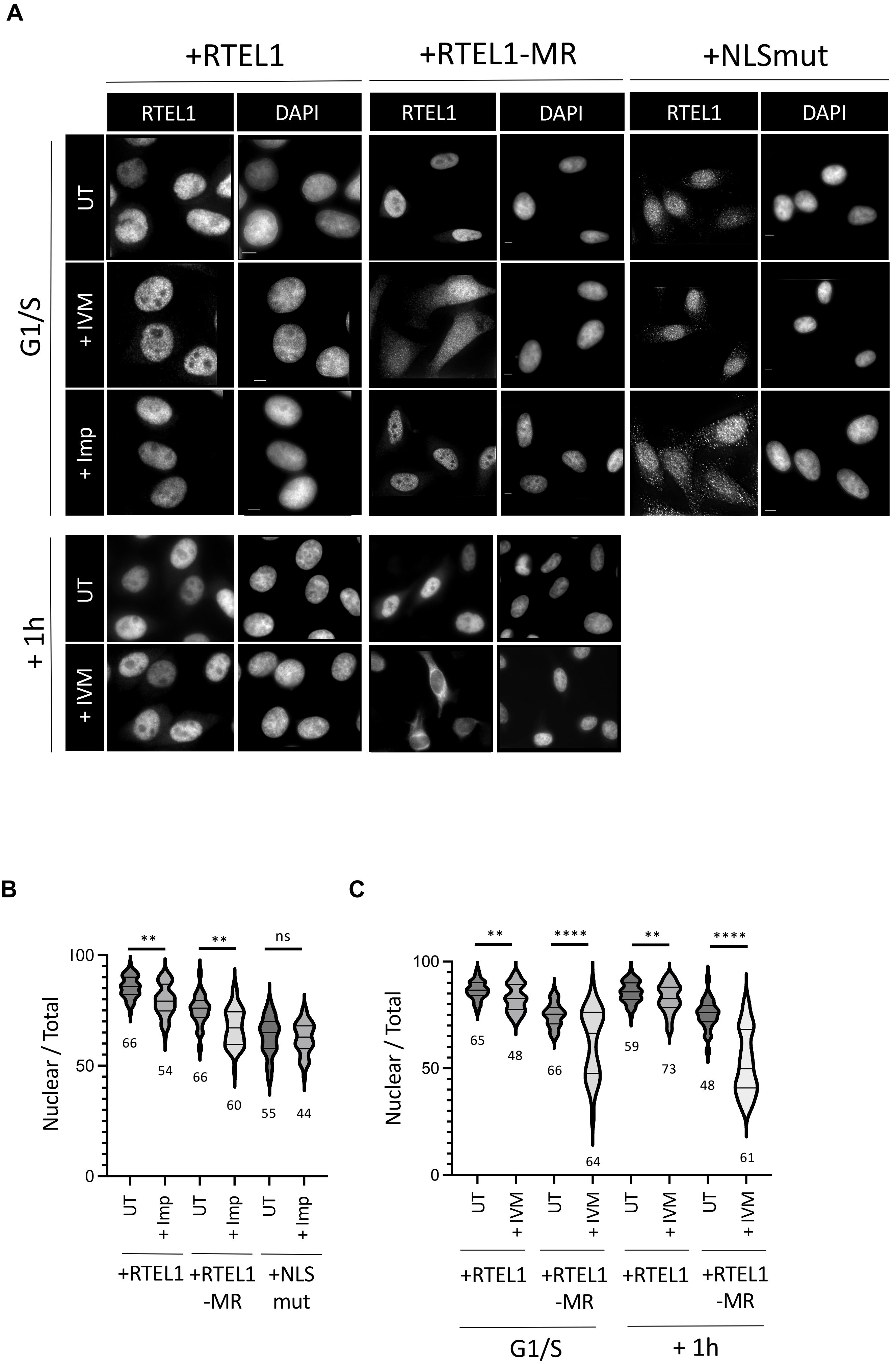

Supplementary Figure 3

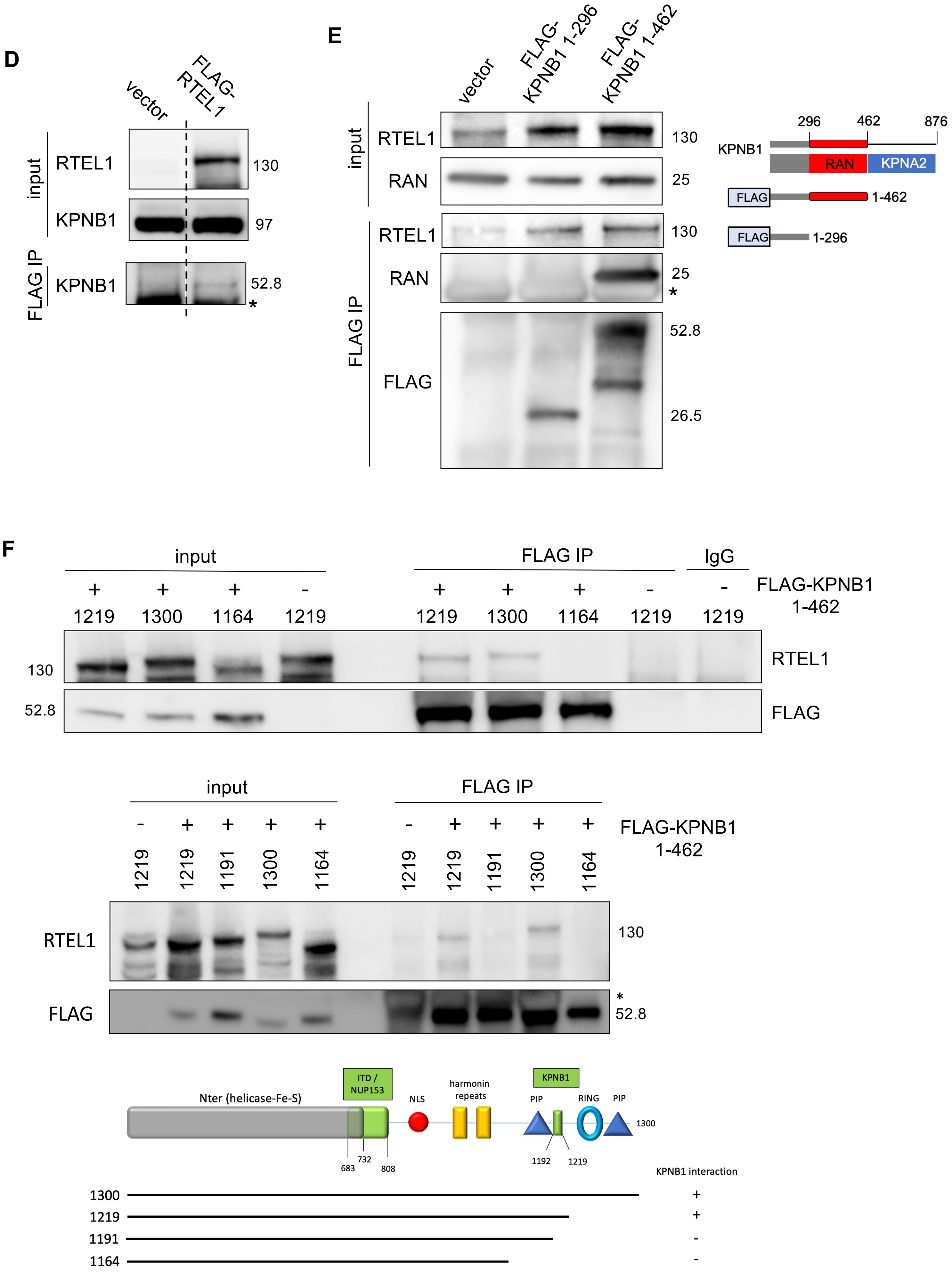

Supplementary Figure S4

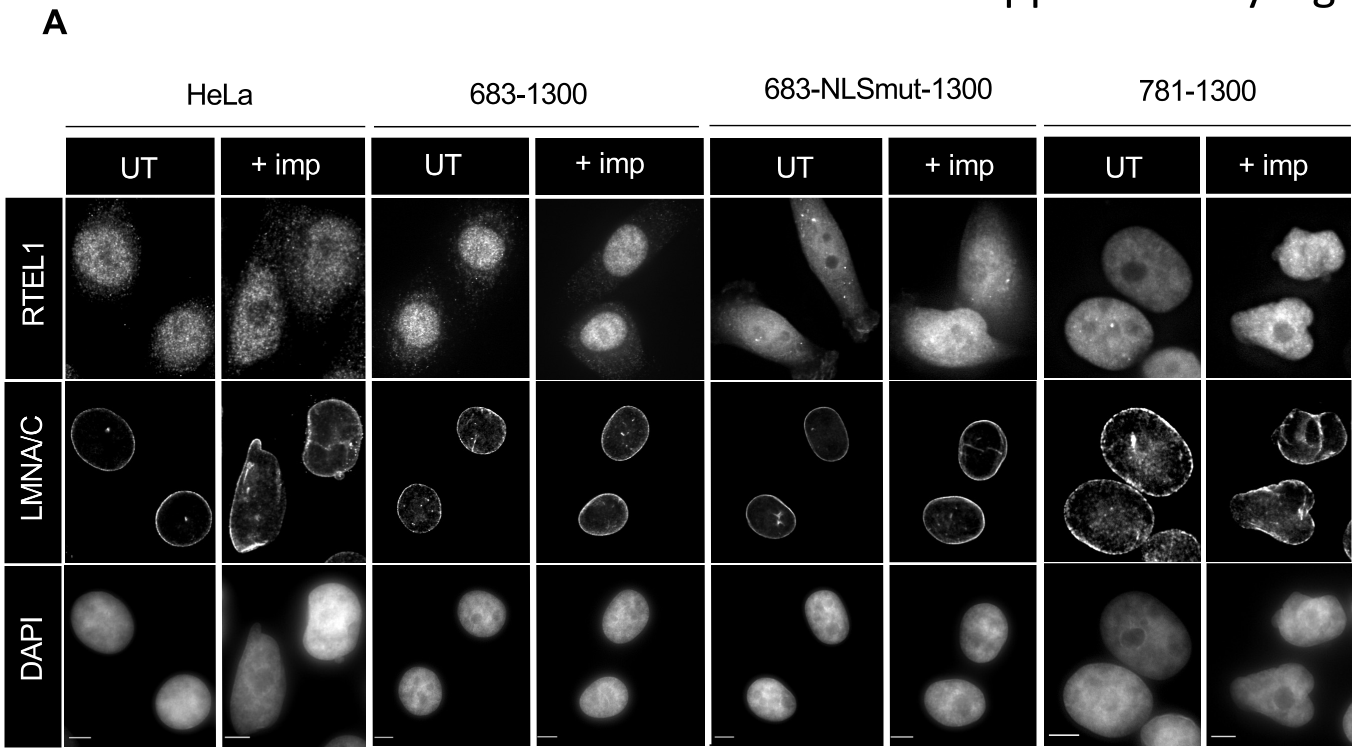

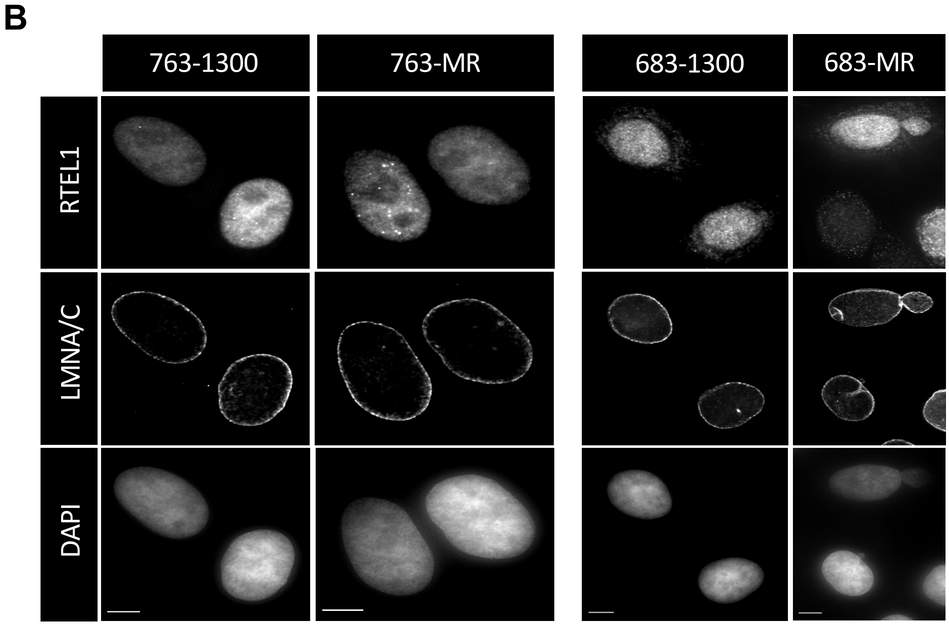

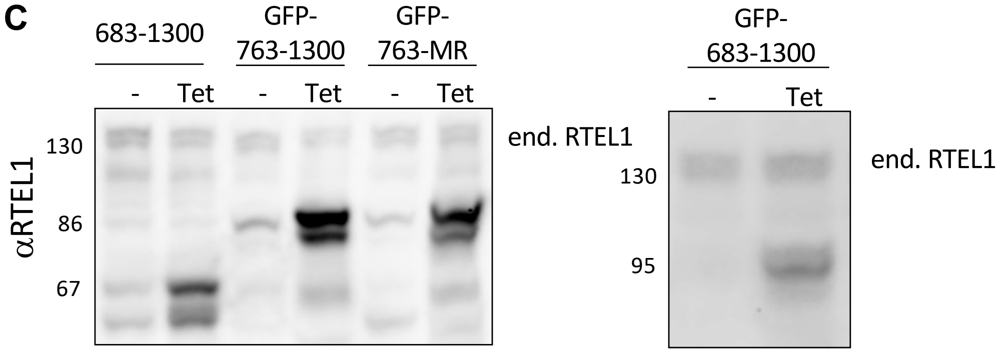

Supplementary Figure S4

D

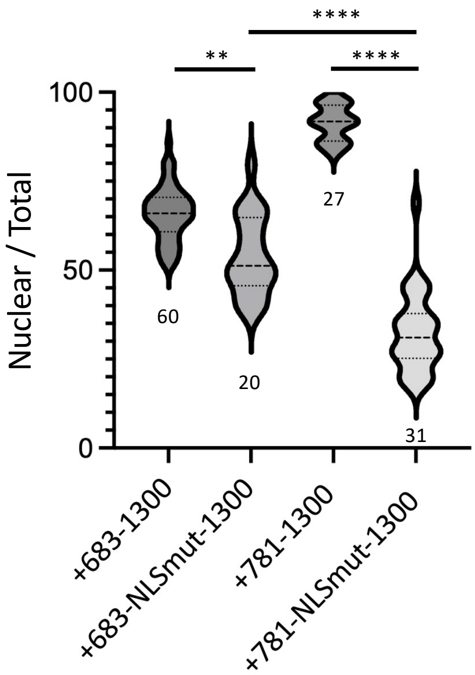

E

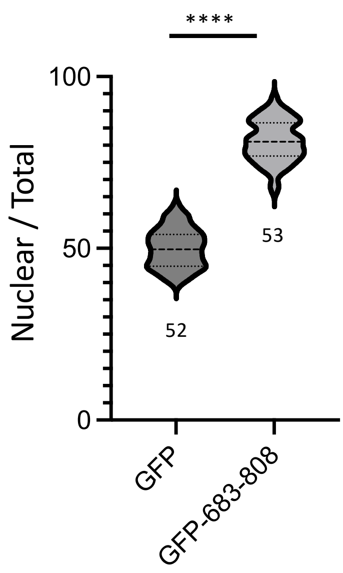

F

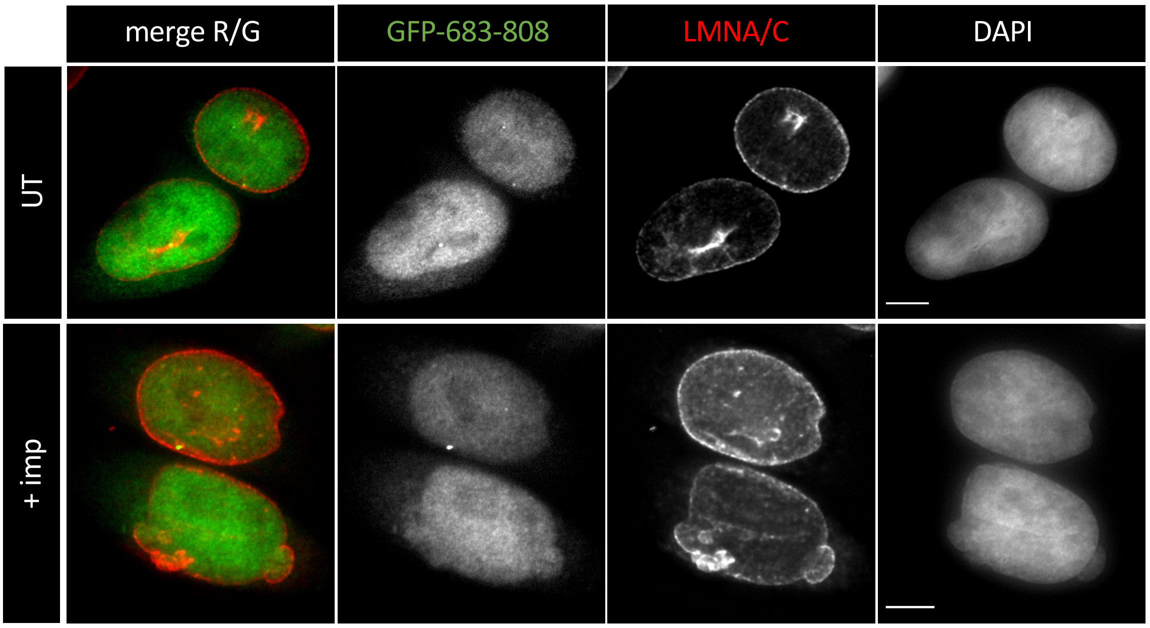

Supplementary Figure S5

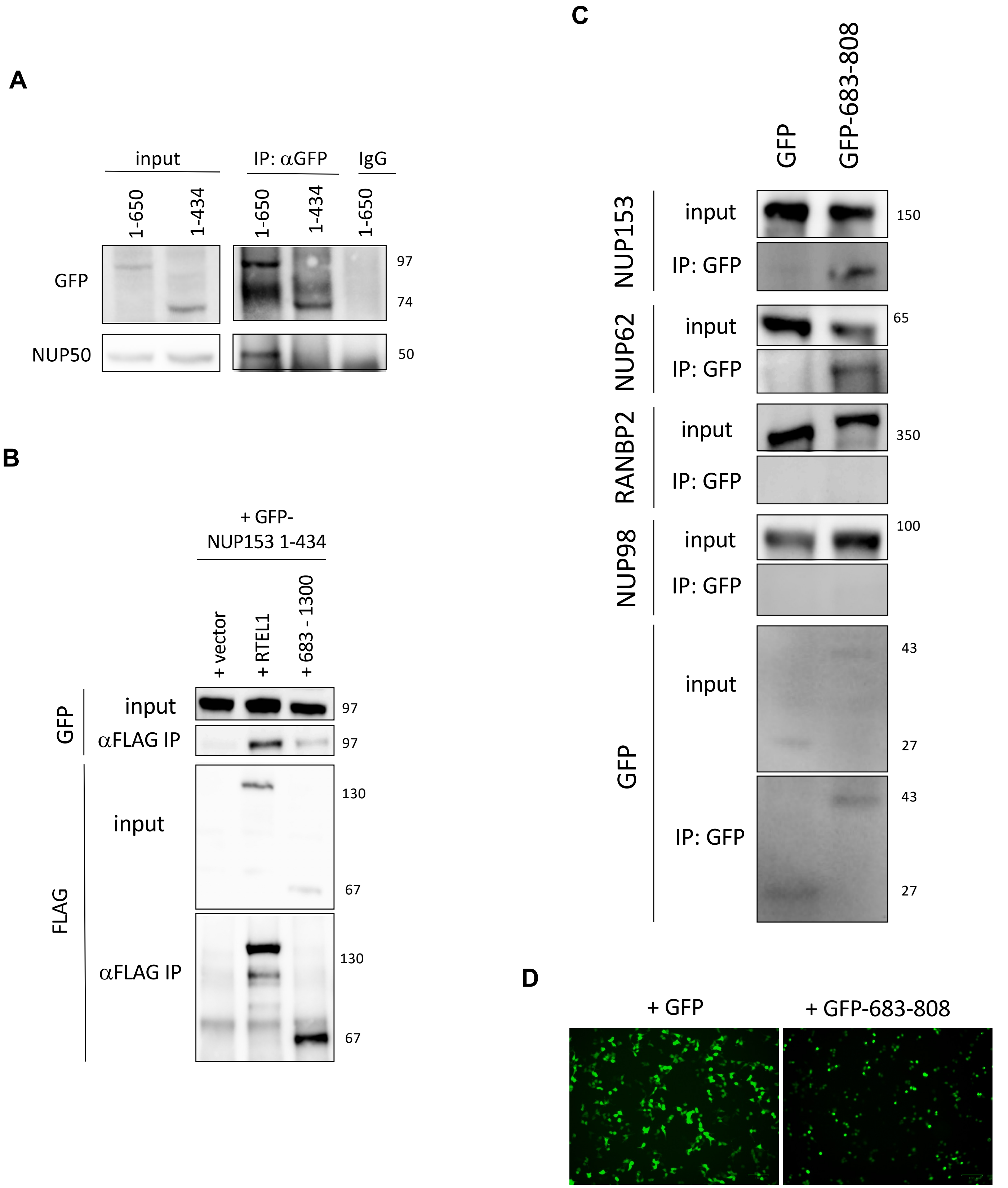

Supplementary Figure 6

A

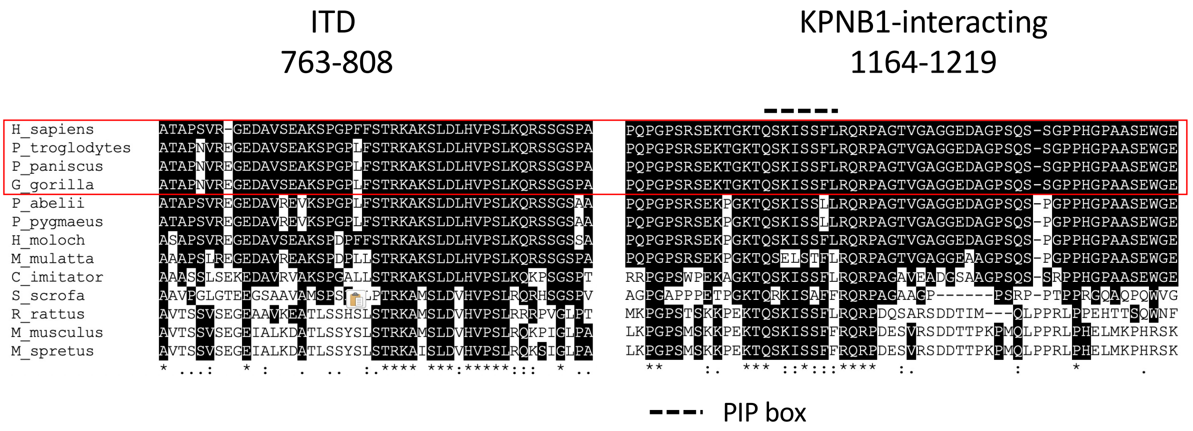

Supplement: Supplementary file 1 [file cells-12-02798-s001.zip › cells-2733278-supplementary.pdf]
